# Walking in a Patient’s Shoes: An Evaluation Study of Immersive Learning Using a Digital Training Intervention

**DOI:** 10.3389/fpsyg.2018.02124

**Published:** 2018-11-12

**Authors:** Candida Halton, Tina Cartwright

**Affiliations:** School of Social Sciences, University of Westminster, London, United Kingdom

**Keywords:** inflammatory bowel disease (IBD), human–media interaction, immersive learning, digital intervention, empathy, patient connectivity, job role, mixed methods

## Abstract

**Objectives:** Evidence suggests that immersive learning increases empathy and understanding of the patient experience of illness. This study evaluated a digital training intervention ‘In Their Shoes’ which immerses participants in the experience of living with inflammatory bowel disease (IBD), highlighting the biopsychosocial impact. The simulation program uses a mobile application to deliver time-based tasks and challenges over 36 h, supplemented with telephone role-play and ‘kit’ items to open and use. This study investigated changes in IBD understanding and connection to patients, empathy and perception of job value in a group of pharmaceutical employees. Additionally, it explored experiences and impact of taking part in the intervention.

**Methods:** A mixed methods pre–post design was utilized, with an opportunity sample of employees taking part in the training. 104 participants from sites in 12 countries completed measures at baseline and 97 post-intervention. Measures included the Toronto Empathy Questionnaire, Prosocial Job Characteristics Scale, and structured questions around IBD understanding and connection to patients. Two focus groups (*N* = 14) were conducted regarding participants experiences of the intervention to complement an open-response question in the questionnaire (*N* = 75). Qualitative data was analyzed using thematic analysis.

**Results:** Following the intervention, there were statistically significant increases in IBD understanding and connection to patients (*p* < 0.00025), evaluation of organizational innovation (*p* < 0.00025), empathy (*d* = 0.45) and prosocial job perceptions (*d* = 0.28). Qualitative analysis revealed more fully the transformative personal journey undertaken by participants which provided ‘eye opening’ insight into the psychosocial impact of living and working with IBD. This insight encouraged patient perspective-taking and a strong desire to promote patient advocacy and reduce stigma around chronic illness. Finally, greater organizational pride and connectivity was evident for some participants.

**Conclusions:** An immersive training program, focussing on the lived experience of illness, led to significant increases in disease understanding and empathy. These findings align with other literature evaluating immersive learning and the potential for increasing knowledge, empathy and motivation. The present study offers opportunities to extend this outside of the body of work focussing on healthcare practitioners and explores the benefits of using this type of learning experience within an organizational setting.

## Introduction

Immersive learning involves placing individuals into scenarios, either physically or virtually, to teach knowledge as well as practice techniques and skills. The learner may undertake the task as themselves or may become someone else, ‘walking in the shoes’ of another person experiencing a different point of view. Immersive learning and simulation experiences which deliver this perspective-taking are many and varied: attending a theatrical performance with structured discussion (Shapiro and Hunt, 2003), inhabiting a virtual game world (Ke, 2016), or putting on a pair of virtual reality goggles to transport you to a specific location or particular perspective (Hansen, 2008). Research evidences the impact of these novel learning interventions across different age groups and disciplines. In primary schools to reduce bullying (Sapouna et al., 2010); in Universities to promote social empathy (Nickols and Nielsen, 2011), policy learnings (Kelley and Johnston, 2016) and global citizenship ‘REAL LIVES’ (Bachen et al., 2012) and in workplace settings to enhance customer engagement skills (Saini et al., 2017). Immersive learning is also an established and popular tool in healthcare. Interventions aim to increase understanding of patienthood, the impact of living with a chronic or acute illness and to enhance empathy. The present study concerns a digital training intervention ‘In Their Shoes’ which uses immersive learning and simulation techniques to replicate the experience of living with a chronic condition for employees of a pharmaceutical company.

### Experiential and Immersive Learning

Kolb’s (1984) experiential learning cycle describes the process “whereby knowledge is created through the transformation of experience” (Kolb, 1984, p. 38). In a four-stage process, Kolb outlines how an individual moves through: (1) concrete experience, (2) reflective observation, (3) abstract conceptualization and (4) active experimentation. The individual ‘grasps’ the information, taking it in and then ‘transforms’ it as they interpret and act. Immersive and simulation learning interventions, especially those that require the learner to take on a role, facilitate experiential learning by providing the opportunity to actively experiment, interpret and act on knowledge gained during a single learning experience. A common objective of immersive learning is to enhance empathy, which can be described as having two inter-related dimensions: cognitive and affective (Preston and de Waal, 2002). This could be taken to align with the experiential learning cycle: cognitive empathy measuring the skills-based aspect of learning, where a person is able to recognize and understand another’s experience (Kourakos et al., 2018). Then affective empathy linking to the transformative aspect of the learning cycle, where the understanding resonates emotionally with the individual and they start to be able to interpret their knowledge, exploring concepts beyond the facts they are presented with (Kourakos et al., 2018). Batt-Rawden et al. (2013) note that healthcare training interventions traditionally target the cognitive empathy component for modification because it can be considered a skill, whereas some authors argue that affective empathy is regarded as a personal trait, which lies beyond the scope of teaching (Cuff et al., 2016).

### Empathy Within Healthcare Professional Training

Connecting with ‘patienthood,’ the experience of living with an illness (Breathnach and Kelly, 2016), is a common objective for healthcare professional (HCP) training, in order to enhance understanding of the patient perspective. Increasing HCP empathy is of interest as this is demonstrated to positively impact the therapeutic alliance, leading to increased patient satisfaction (Kim et al., 2004), adherence (Di Blasi et al., 2001), and improved health outcomes (Hojat et al., 2011; Rakel et al., 2011; Canale et al., 2012; Roche and Harmon, 2017). As Derksen et al. (2013) found in their systematic review, empathy lowers patient anxiety and distress and delivers significantly better clinical outcomes for patients. However, enhancing empathy for the benefit of the HCPs themselves (rather than their patient) is not without controversy (Pedersen, 2009). Wilkinson et al. (2017) note in their systematic review the conflicting evidence for whether enhanced empathy protects against burnout or increases its likelihood. Nevertheless, empathy interventions and their impact for HCP training is a well-studied area. Recent reviews include simulation as a methodology for teaching empathy to health professional students (Bearman et al., 2015) as well as two reviews focusing on empathy interventions more generally (not limited to simulation) for medical students (Batt-Rawden et al., 2013) and nurses (Brunero et al., 2010). However, as the review authors note the variety and complexity of empathy related simulations, and variety of their measures, make definitive conclusions about effective design and evaluation challenging.

Examples in the literature where a healthy learner vicariously experiences illness use a variety of simulations to create the experience. Corr et al. (2017) simulate living with melanoma by requiring participants to wear a temporary tattoo. Eymard et al. (2010) use a variety of physical items to replicate the disability of older age such as a physical limitations suit, COPD lung simulator and glasses that simulate age-related sight conditions such as glaucoma and macular degeneration. Bunn and Terpstra (2009) used a 40-min recording to simulate an auditory hallucination associated with mental illness. Tong et al. (2017) used an avatar to experience the restrictions of chronic pain on daily life activities. Other studies have focused on pill burden or dietary adjustments associated with chronic illness, requiring participants to adhere to medication (Welborn and Duncan, 1980; Pinkston, 2009) or dietary regimens (Whitley, 2012; Harris et al., 2018). One study with student nurses placed them in a hospital bed for 4 h to experience the lack of agency as a bed-bound inpatient. With the exception of Tong et al. (2017), where the intervention was designed to help care-givers access the experience of living with chronic pain, programs reported in the literature are designed for HCP training.

### Characteristics of Empathy Interventions

Despite the heterogenous nature of evaluation studies, some key characteristics of successful interventions of patient perspective-taking do emerge. In their review of 17 nursing empathy interventions, Brunero et al. (2010) found 11 which reported statistically significant improvements in empathy – noting that experiential styles of learning offered most promise. Similarly, Bearman et al. (2015) review of 27 HCP educational interventions found that those where the student was required to ‘be’ a patient, for some or all of the intervention, were more successful in increasing empathy. In contrast, a nursing education intervention to build empathy for patients with acquired brain injury found higher post-intervention empathy scores for those taking the role of the rehabilitation nurse not the patient (Levett-Jones et al., 2017).

Duration of simulated learning programs vary. A single short intervention – for example the well-established ‘Aging Game’ which takes place in a single 90 min session (McVey et al., 1989; Varkey et al., 2006). For this, students receive a series of props to simulate common physical disabilities of aging (sight loss, poor motor skills etc.) and then have to complete a role-play at five different ‘stations’ simulating different healthcare encounters. Other simulation training is structured as an entire module, integrated into medical school curricula (Rosenthal et al., 2011). There are fewer descriptions of interventions where the simulation is merged into a daily routine. Harris et al. (2018) evaluated the impact of pharmacy students planning for and following a 3-day diet plan for a patient with diabetes to enhance empathy and counseling confidence; Corr et al. (2017) had medical students wear a melanoma tattoo in a prominent place for 24 h. In both studies, the authors attribute this durational aspect as central to the intervention’s success in promoting empathetic perspective-taking, noting that as the simulation moved out of the classroom and into daily social interactions, the reality of the impact of patienthood is further revealed. Another attribute of the Corr et al. (2017), study was the authenticity of the simulation where the tattoo was a direct copy of an actual patient’s melanoma. Authenticity, credibility and attributable scenarios have been found to support participant engagement more widely, for example Tong et al. (2017) where the authors designed a pain simulation game ‘AS IF’ using a chronic pain patient as a narrator to a series of challenges.

Interestingly, only one of the empathy interventions included in the Brunero et al. (2010); Batt-Rawden et al. (2013) or Bearman et al. (2015) systematic reviews involved digital delivery [Bunn and Terpstra (2009) auditory hallucination]. However, there are examples of empathy intervention design borrowing devices from the gaming discipline. This includes employing a central narrative, peaks of intensity, branching and aspects of what the serious gaming literature classifies as ‘adventure’ (overcoming obstacles); ‘strategy’ (planning) and ‘role-play’ (Boyle et al., 2016; Ke, 2016). Other characteristics of serious gaming are not present, for example reward mechanisms for persistence or competition between ‘players.’ The use of avatars within computer-based simulation games for healthcare teaching is evident (Norris et al., 2013, 2014), although not reported specifically for empathy.

### Empathy Beyond the Healthcare Environment

There are fewer user or beneficiary perspective-taking interventions documented outside the healthcare setting, although some evidence for benefits does emerge. Work-place psychology literature shows a relationship between perceived role value and job performance: feeling that your role ‘does good’ motivates you to do it better. In a sales environment, this concept is developed by Grant (2008) who describes how pro-social behaviors (a behavior intended to help another), increased following contact with beneficiaries and led to improved job performance and customer (beneficiary) orientation. Grant (2008) and Saini et al. (2017) link customer-orientation and pro-social behaviors to job satisfaction, organizational commitment, engagement and motivation. Parallels might be drawn with findings from the healthcare domain where empathetic physicians are less likely to experience burnout and compassion fatigue (Brazeau et al., 2010; Gleichgerrcht and Decety, 2013). Empathy has also been linked to design thinking and innovation (Brown, 2009; Kolko, 2015). Design Thinking describes a process for problem solving and product design which uses experimentation and rapid prototyping (Razzouk and Shute, 2012; Roberts et al., 2016), which has at its core a focus on user experience, and a belief that building ‘user-empathy’ is the critical first step for innovation and problem solving. Examples in the literature include Johnson et al. (2014) who conducted an experiment which demonstrated that participants who engaged in empathetic experiences produced concepts (for an alarm clock or litter collection device) with significantly higher rates of original product user interaction features than those who did not. Gamman et al. (2012) asked designers to ‘Think Thief’ in order to inspire and help them design against crime – for example in strategies to prevent bike or bag theft. Research into effective leadership highlights the importance of empathy skills, particularly for leaders working in global organizations (Walumbwa et al., 2008).

### Disease Simulation for Pharmaceutical Company Employees

Immersive learning thus offers opportunity to deepen understanding of patienthood, with implications for employee and organizational benefits beyond healthcare practice. Regular training for pharmaceutical employees, including those who are not in a medical role, covers basic epidemiology and disease pathology for therapy areas of relevance to the company product portfolio. Created by Takeda Pharmaceuticals, with development partner The Method, In Their Shoes^®^ is designed to enable employees to ‘live’ the life of a person with a chronic condition, specifically inflammatory bowel disease (IBD). The intention of the immersive training program was to enhance understanding of the disease, engage and motivate employees in their role, as well as challenge them to think differently about solutions for people living with IBD.

Inflammatory bowel disease encompasses ulcerative colitis (UC) and Crohn’s disease (CD), which are characterized by chronic gastrointestinal inflammation. Typical symptoms include diarrhea, fatigue, abdominal pain and cramping, blood in stools, reduced appetite and weight loss. The impact of IBD on patient quality of life is well documented (De Rooy et al., 2001; Rochelle and Fidler, 2013; Matini and Ogden, 2015; Palant et al., 2015). This impact is influenced not only by physical symptoms but also by psychosocial factors. The course of these diseases is unpredictable, and the treatment of IBD focuses on the maintenance of remission and treatment of relapse (Goldring et al., 2002). IBD prevalence is on the rise, with estimates placing prevalence at around 0.5–1% (Molodecky et al., 2012; Ghosh and Premchand, 2015), with diagnosis typically in early adulthood (Johnston and Logan, 2008). Amiot and Peyrin-Biroulet (2015) note that despite intensive research, IBD remains incompletely understood.

Given the relative paucity of research in the workplace context, we were interested in the process and experience of a digital immersive program to simulate patienthood, as well as its impact on outcomes with potential value for the organizational setting. The present study therefore aimed to investigate the impact of an immersive training program on pharmaceutical employees’ understanding of IBD and connection to patients, empathy, and perception of job value. We hypothesized significant increases in disease understanding, empathy and prosocial job perceptions pre–post program. Additionally, we employed qualitative methods to explore employees’ experiences and perceptions of taking part in the intervention.

## Materials and Methods

### Design

The evaluation used a mixed methods design with an opportunity sample of pharmaceutical employees taking part in the training intervention ‘In Their Shoes’ between September 2017 and February 2018. A mixed methods approach is particularly valuable in this context to illuminate both the processes and effects of a novel intervention (Abildgaard et al., 2016). An online questionnaire delivered via Qualtrics was administered at baseline and post-intervention. Additionally, two focus groups with selected study participants took place in the United Kingdom and France post-program to explore in-depth experiences of taking part in the intervention. All those indicating a willingness to take part in a focus group conducted in the English language were invited to take part (from United Kingdom and French offices). Ethics approval for the evaluation was obtained from the University of Westminster Ethics Committee.

### Participants

**FIGURE 1:**
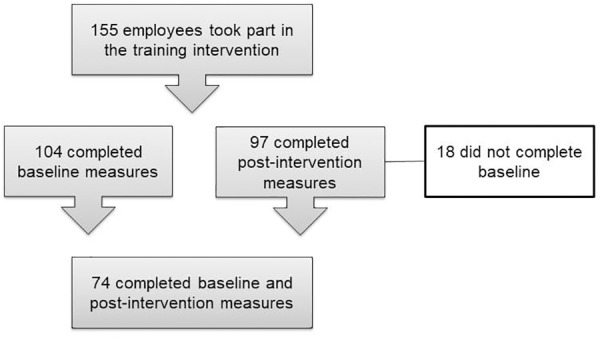
Participant flow.

One hundred and fifty five employees of a pharmaceutical company took part in the immersive program which is run periodically in Takeda offices in Europe, Asia, North and South America. Recruitment to the program followed standard company procedure whereby staff either volunteered to take part, or teams/key personnel were nominated by managers. All those taking part were invited to participate in the evaluation. One hundred and four participants from 12 countries completed baseline measures (67% response rate), 97 completed post-intervention measures. Seventy four completed both baseline and post-intervention questionnaires (71% response rate). Participant flow is presented in Figure 1.

At baseline, there were 66 females and 37 males, with a mean age of 40.6 years (*SD* = 8.85, range 20–62 years). Virtually all (97%) were educated to at least degree level, with 43% having postgraduate qualifications. The majority of participants (56%) had been with the company for less than 3 years, 26% for 3–10 years, and 16% for more than 10 years. Twenty (19%) were registered HCPs, but the majority (88%) were not currently practicing. Only five (4.8%) had taken part in an immersive training program previously. Over a third (36.5%) had personal experience of chronic disease (self, close family, or friend).

### The Intervention

The design of In Their Shoes^®^ draws on the biopsychosocial model of illness (Engel, 1977), highlighting the psychological and social impact of living with a chronic condition as well as physical symptoms. The 36 h constructed narrative contains around 70 individual challenges typically faced by someone living with Inflammatory Bowel Disease. As well as simple directions (“go immediately to the toilet”) it also creates scenarios and jeopardy where the individual is required to ‘choose’ between their disease or their life: (“you have left your medication at home, do you hope you’ll be ok, or risk missing an important business meeting to return and collect?”). Scenarios touch on the stresses chronic illness exacts on work and personal relationships. To add urgency and monitor engagement, certain challenges are timed and require the participant to send a photograph to document that they have completed the task. Participants are not incentivized to complete the tasks and there is no competitive element between ‘players.’ However, some challenges include text from real IBD patients commenting on when they faced a similar scenario.

The immersive simulation is primarily delivered through a smartphone application. The participants download this and read a standardized patient profile detailing basic disease state information and time since diagnosis, there is also dietary guidance. The application has an avatar (head and shoulders only) with basic personalization (hair/eye color; skin tone; face shape; hair style; glasses/facial hair; shirt color and style). The avatar is present on the initial application screen and shows signs of stress and illness during the more intense narrative sections (e.g., sweating, turning red or pale). At four points during the experience there are live role-plays (via phone) with an actor following a specific script. In the first, a ‘nurse’ uses the Bristol Stool Chart (Heaton and Lewis, 1997) to classify the participant’s stool (feces) and describes how to give a stool sample in preparation for an upcoming clinic appointment. In the second the ‘boss’ invites the participants to a high level meeting – which requires long-haul travel; the third involves a friend frustrated that the participant will miss an important social engagement. In the fourth a ‘surgeon’ calls to discuss the risks and benefits of stoma surgery. Participants are given a kit of wrapped items which they keep with them during the experience, this contains 12 items which underscore challenges (e.g., blood capsule to simulate blood in stool; stool sample pot – used during nurse role-play; protective bed sheet for those participating in the optional night experience). The narrative structure and challenges that occur during the immersive intervention draw from published literature on the patient experience of IBD (e.g., Palant et al., 2015) and input from patients and patient groups.

### Measures

#### Demographic Information

Demographic information was collected at baseline only: age, gender, education, nationality, length of service, current company role and personal experience of chronic disease (self/family/friends).

#### IBD Understanding and Connection to Patients

Questions were adapted from Shapiro and Hunt (2003) which measured disease specific understanding following a theatrical educational intervention. The seven questions were rated on a 7-point likert scale from ‘not at all’ (1) to ‘very much’ (7). They assessed understanding of IBD and its impact, empathy toward people with IBD, confidence in talking about IBD, and degree of connection with patients. An additional question assessed perceptions of organizational innovation.

#### Impact of Evaluation

Two questions were included in the post-intervention questionnaire to assess perceived impact of the intervention (confidence in incorporating insights into work role; gaining a valuable, new perspective on how patients’ live with IBD). A further question asked participants to rank the four elements of the program (text messages, role play, avatar, kit items) from most to least impactful (1–4).

#### Toronto Empathy Questionnaire

Toronto Empathy Questionnaire (Spreng et al., 2009) consisting of 16 questions, rated on a 5-point scale, from ‘never’ (0) to ‘often’ (4). Half the items are negatively phrased and reverse scored. Scores are summed to derive total empathy score (0–64). In the present study, Cronbach’s alpha was 0.86 at baseline.

#### Prosocial Job Characteristics Scale (PSJC) (Grant, 2008)

A self-report measure of the extent to which jobs provide opportunities to impact on others, rated on a 7-point scale from not at all (1) to very much (7). Whilst the original PSJC contains two sub-scales (impact and contact), only the first was included here as it was deemed most relevant to the current study, a nine-item subscale ‘Job opportunities for impact on beneficiaries’ (Cronbach’s alpha 0.96). This incorporates three further subscales: frequency of positive impact on others, magnitude, and scope.

### Procedure

Participants were notified of the evaluation study during the normal registration process for ‘In Their Shoes^®^’. The study was open to all and participation was voluntary. A Participant Information Sheet was provided as part of the pre-registration information. The standard briefing meeting (face-to-face or via web conferencing) was extended to allow for a short presentation about the study with open question and answer session (10 min duration in total), led by a member of the study team (CH). For quantitative data collection, a web link was sent to all participants directly after the briefing meeting. This link took participants to a webpage with study information and consent form. If the consent form was completed, participants were then directed to the baseline questionnaire. This had to be completed prior to the start of the intervention. The post-intervention questionnaire link was sent by the study team to all participants directly after the close of the intervention. A debrief meeting at the end of the training period provided an opportunity for duty of care. All responses were collected in English.

For the focus groups, participants from the United Kingdom and France were asked if they were interested in taking part during the debrief session and provided with a Participant Information Sheet. They completed a consent form and were given the opportunity to ask further questions prior to the start of the focus group.

### Qualitative Data Collection

Qualitative data was obtained from two sources: (a) an open question included in the post-intervention questionnaire (please tell us how it felt to take part in the In Their Shoes program) (*N* = 75); (b) two focus group discussions in France and the United Kingdom (*N* = 8 and 6, respectively). Participation in the focus group discussions was voluntary. These took place post- intervention in Takeda offices, lasting approximately 70–90 min. Focus groups were facilitated by the research team (led by TC) and were conducted in English. Topics covered included how it felt to take part in the program, what participants had learnt from the intervention, how they felt toward patients with IBD, any impact on their job role and what elements of the program they felt were most effective. The facilitators encouraged group discussion about experiences and allowed for natural development of topic areas relevant to the group (Parker and Tritter, 2006). Recordings were transcribed by a professional agency and checked by researchers.

### Data Analysis

Statistical data analysis were conducted using SPSS version 23. All statistical analyses comparing baseline and post-intervention scores are based on the 74 complete datasets. Non-parametric tests (Mann–Whitney-*U*, Wilcoxon signed rank, Chi square, as appropriate) were used to compare differences between those who did/did not complete on baseline measures, and to assess pre–post differences on ordinal level variables (disease understanding and connection to patients). To assess pre–post differences in empathy and prosocial job role, paired *t*-tests were conducted. Independent *t*-tests were conducted to assess group differences in primary outcome variables (e.g., gender). Effect sizes for scale changes were calculated using Cohen’s *d*, with effect sizes defined as small (*d* = 0.2) and medium (*d* = 0.5) (Cohen, 1988).

Qualitative data (responses to the open ended questions from the post-intervention questionnaire and focus group transcripts) were analyzed inductively using thematic analysis as outlined by Braun and Clarke (2006). An experiential or realist approach was adopted to identify patterns in participants’ accounts of their experiences of taking part in the intervention (Braun and Clarke, 2006). The second author (TC), an experienced qualitative researcher, read through the open response data set several times and undertook detailed coding, noting key words and sections of text. In the second stage, this was further refined to develop an initial list of codes and broader themes that represented recurrent patterns in the data and discussed collaboratively with the second author (CH). Analysis of focus groups was based on codes from stage two of the open response analysis and further expanded to add new codes and further interpretative detail. Then a list of key themes and illustrative quotes relating to participants’ experience of the intervention was compiled by TC. At this stage, CH independently coded the focus groups and both authors compared coding frameworks to debate and arrive at a final thematic structure for the whole data set. Quotes from both data sets are used to illustrate the findings and reflect responses across countries, notation is used to indicate when quotes are derived from focus groups (FG).

## Results

There were no statistically significant differences between post intervention completers and non-completers on demographic or study variables at baseline.

### IBD Understanding and Connectivity

As hypothesized, there were significant increases in reported IBD understanding and connectivity to patients post-intervention (see Table 1). All seven questions showed statistically significant change: understanding of living with IBD (*z* = 7.475), understanding of physical symptoms of IBD (*z* = 6.197), understanding of emotional and psychological issues (*z* = 5.867), empathy toward people with IBD (*z* = 6.008), confidence talking to stakeholders about the impact of IBD on patients’ lives (*z* = 6.045), and connection to patients (*z* = 5.607). There was also an increase in perceptions of organizational innovation in its approach to patient-centered care (*z* = 3.687, *N*-Ties = 46, *p* < 0.00025).

**Table 1:** Disease understanding and connectivity descriptives, at baseline and post-intervention.

Item	Baseline median (interquartile range)		Post-intervention median (interquartile range)	
Understanding of:				
IBD	4.0	(3.0–5.0)	6.0^∗^	(5.0–6.0)
Physical symptoms	4.0	(4.0–5.0)	6.0^∗^	(5.0–6.0)
Psycho-emotional issues	4.0	(3.0–5.0)	6.0^∗^	(5.0–6.0)
Empathy for people with IBD	5.0	(4.0–7.0)	7.0^∗^	(5.0–7.0)
Confidence in talking about IBD	4.0	(3.0–6.0)	6.0^∗^	(5.75–7.0)
Connected to patients	4.5	(4.0–5.0)	6.0^∗^	(5.0–6.0)
Organizational innovation	5.0	(4.0–7.0)	6.0^∗^	(5.0–7.0)
**Post-intervention only variables**				(6.5-7.0)
ITS offers new perspective on IBD			7.0	(6.5–7.0)
Able to incorporate insights of ITS			6.0	(5.0–7.0)

*^∗^Statistically significantly different from baseline (p < 0.00025), 1-tailed.*

Evaluating the value of the program, the intervention was highly ranked in terms of providing a valued perspective on living with IBD and participants felt confident in incorporating these insights into their role (see Table 1).

### Empathy and Prosocial Job Perceptions

As hypothesized, there was a statistically significant increase in empathy post-intervention, compared with baseline (see Table 2), with a medium effect size (*d* = 0.45). There were small but statistically significant increases in prosocial job perceptions, both for the overall scale (*d* = 0.28) and three subscales: frequency of positive impact on others (*d* = 0.33); magnitude of positive impact (*d* = 0.2), and scope of positive impact (*d* = 0.19).

**Table 2:** Descriptives for empathy and prosocial job perceptions measures, baseline and post-intervention.

	Baseline mean (*SD*)	Post-intervention mean (*SD*)	95% confidence interval	*t*	df	*p*-value^∗^
Empathy	43.51 (8.44)	46.88 (6.62)	1.24–5.47	3.167	73	0.002
Job role	4.84 (1.12)	5.13 (1.00)	0.10–0.49	2.984	71	0.002
Frequency	4.61 (1.17)	4.98 (1.06)	0.15–0.58	3.396	71	0.0005
Magnitude	4.99 (1.23)	5.25 (1.08)	0.02–0.50	2.157	71	0.017
Scope	5.00 (1.18)	5.21 (1.04)	0.02–0.44	1.821	71	0.037

*^∗^1-tailed*.

There were no statistically significant differences in empathy or prosocial job perceptions change scores by gender, previous experience of chronic illness, or whether participants were HCPs.

### Evaluation of Program Elements

Median scores indicated that the text messages and role plays were ranked most highly, with the majority ranking these elements in the first two categories (70 and 72%, respectively). IBD kit items were ranked third, with the avatar rated as the least impactful (see Table 3).

**Table 3:** Ranking of program elements (%).

	Most impactful 1	2	3	Least impactful 4	Median score
Text messages	37.2	32.6	26.7	3.5	2
Role plays	43.0	29.1	11.6	16.3	2
IBD kit items	15.1	30.2	47.7	7.0	2.5
Avatar	4.7	8.1	14.0	73.3	4

### Qualitative Findings: Experiences of Taking Part in the Immersive Program

**FIGURE 2:**
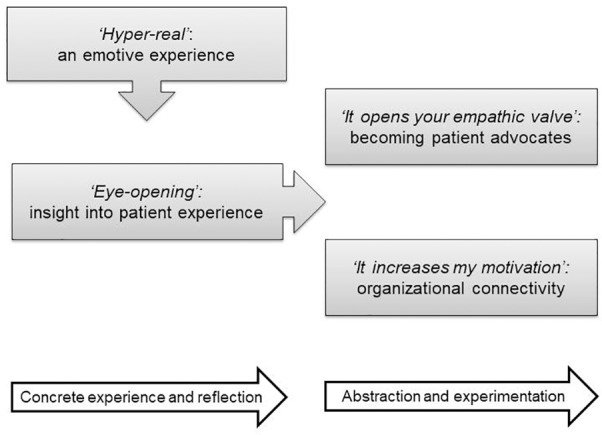
Overview of qualitative themes.

Four core themes were identified from the qualitative data (see Figure 2 for overview of themes). The first, *A ‘hyper-real’ and emotive experience*, describes the physical and emotional intensity of taking part, which enabled personal *insight into the patient experience* as described in the second theme. The emotional engagement and understanding gained from the program was described in the third theme as building empathy toward patients resulting in a desire to increase awareness *(‘It opens your empathic valve’: Becoming patient advocates*). The final theme outlines the increase in *organizational connectivity* described by some participants through greater appreciation of their role in patient care. Together the themes appear to reflect an experiential journey, consistent with Kolb’s learning cycle, in which participants build their understanding of the disease, reflect on the real-life impact and then deepen their reflections as they interpret and act on their new insight. This provides a new, critical, perspective on the wider implications of living with a chronic condition.

#### A “Hyper-Real” and Emotive Experience

“Initially there was this “bit of fun” element to it, but by the time that I had the phone call from the doctor I felt fully immersed in the experience, and actually felt quite emotional about being told that I’d need the operation - and I don’t have IBD.” (United Kingdom)

All participants described the intensity of taking part in “a brutally realistic experience” that was stressful and intrusive and yet overwhelmingly a positive experience: “It was a great experience, I felt sick, lonely and sad” (Austria). The rollercoaster of emotions experienced during the program facilitated a strong connection to the reality of people’s experiences of living with IBD. Commonly described were sadness and distress, anger and frustration, fear and anxiety, guilt (when a task was missed), shame and embarrassment, venting (“I want a doughnut!”), all of which impacted on mood (“you feel pretty low by the end of it all,” United Kingdom). It was this depth of emotional engagement that often led participants to recognize the effectiveness of the program. Participants described feeling physically and emotionally exhausted and found managing the conflict between the demands of work and illness particularly stressful, with many discussing lost productivity (“I found it impossible to combine IBD flare period with my job,” Czechia). The experience stimulated reflection on the processes involved at managing different phases of the disease, with one participant describing how he felt “completely lost in the middle of the jungle.”

Such personal and visceral experiences were seen to result in deeper impact (“you don’t simply return to your life,” United Kingdom) in comparison to other educational programs: “you have to ask yourself questions…you can’t feel that In Their Shoes is a game” (France, FG). There were individual differences with regard to which elements of the program were most impactful, nevertheless for all participants it was the package of elements (“the richness of the application”) that made it particularly powerful. The frequency and duration of toilet visits were frequently mentioned challenges along with the emotional impact of the role plays (“I cried when I got the call from my Doctor saying surgery was the next step”). Kit items were often discussed in relation to impact on self and others, for example, one participant described feeling “like I’d aged 30 years”, whilst another joked that his wife got “a bit of a shock” when using the paper pants/bed cover. Although the avatar produced mixed responses, for some it enabled stronger emotional connection, “almost like you’re looking in a type of mirror” (United Kingdom). Only one participant mentioned dislike of the “gamification style” of the program.

Several described how the experience had caused them to appreciate their own health (“I feel blessed for being healthy,” Croatia) and led to reprioritization “it changes my perspective of things that I do on a regular basis” (UK FG). Thus, despite the discomfort of taking part in the simulation, the personal insight was deemed transformative: “I’ll never be the same” (France).

#### ‘Eye Opening’ Insight Into Patient Experience

“The In Their Shoes program was very eye opening for me to understand how someone with IBD needs to manage not only the treatment of their disease but the other aspects of their life. This disease, and many others, proved to be very disruptive and I have an understanding on how IBD not only causes discomfort and pain but also the tremendous psychological and emotional impact it can have on patients and their loved ones.” (United States)

The physical and emotional challenges faced by participants during the intervention led to a deep-seated appreciation of the psychosocial impact of living with IBD. Participants commonly alluded to the difference between knowledge about the disease and understanding of the patient *experience* enabled by taking part in the program (“a key to get into the life/house of an IBD patient,” Germany). In the focus groups, this led to reflections about the broader implications of living with a chronic condition (‘just imagine if…’) and discussions about stigma, disability rights, and the conflict surrounding decisions to conceal versus acknowledge the disease. For some, “it’s easier to lie than tell the truth” (France, FG) to avoid the shame and potential misunderstanding from others, whereas disclosure was seen as more empowering and facilitative of increasing public awareness by bringing “it out in the open” (United Kingdom). Additionally, impact beyond the individual was widely discussed in focus groups, with many relating how the program had increased awareness in family members, particularly their children: “they found it hard to believe that there were actually people out there that suffer with this every day” (UK FG).

Insight could be categorized into three main areas: loss of control, impact on professional lives, and psychological challenges. The day-to day challenges and frustrations of dietary restrictions (“easier not to eat”) and access to toilets were framed in terms of loss of control: “you realize how many choices it takes away from you” (UK FG). Participants recognized the consequential impact on both social and family life, particularly around the difficulties of planning. Frequent mention was made of the impact of IBD on professional lives, in relation to difficulties in carrying out work roles, curbing ambitions and managing the challenges of commuting – “I probably couldn’t do my job if I had CD” (United Kingdom).

Whilst participants recognized the physical difficulties of IBD – frequent toilet visits, physical pain and sleep disturbance - the greatest focus was given to the psycho-emotional impact of the disease. The demands of the condition was recognized as mentally intrusive, emotionally draining and potentially overwhelming. Focus groups discussed the impact of IBD and other conditions on identity (“it’s like a handicap,” France), with a sense of isolation highlighted as a key vulnerability that should be addressed with access to appropriate support:

“to have someone to speak to, but someone who would be able to understand you as well, and not only someone who can listen to you.” (France FG)

Indeed, it was seen as particularly upsetting when role-play actors lacked understanding, creating a “feeling like no-one cares” (United Kingdom). Lack of public awareness and societal discrimination were perceived as creating further difficulties for people with IBD and other chronic conditions: “those people are totally outside of the society as a lot of things are not adapted to them” (Switzerland).

#### ‘It Opens Your Empathic Valve’: Becoming Patient Advocates

“*I come away with a huge amount of respect for those people that manage their IBD so successfully and feel I am closer to being able to empathize with the patient population.” (United Kingdom)*

This insight into the patient experience – “a window into how patients are feeling” (UK FG) - enabled participants to take on the perspective of those with IBD, at least temporarily, and also connect with the emotional experience of the sufferer.

“We train our compassion very effectively with this exercise and become much more compassionate toward patients with the disease.” (UK FG)

Reflection around the experience of how it feels to live with a health condition extended beyond IBD to incorporate a broader understanding of the challenges of managing long term conditions [“it creates that, or almost encourages that depth of thinking across the board, not just about IBD” (UK FG)]. This more personal connection to the patient experience is illustrated in a discussion around defining patient benefit, reflecting a shift from a paternalistic biomedical perspective to a reprioritization of patient-centered criteria for measuring drug outcomes:

“I think what this exercise made me reflect upon was the fact that we need to, to understand, of course not only the scientific very clear rationale, [of] what we do (in drug development), but we also need to think in the practical daily life, how does it reflect to the patient so that the patient can really have benefit?” (UK FG)

As well as challenging professional assumptions, increased empathy and understanding led to a strong desire for broader action to promote patient advocacy. For example, to increase public awareness (“how do you bring it out in the open, and how do you make people aware of it?” UK FG), reduce stigma and improve access to support.

“Maybe that’s our best advocate for anyone that we see suffering from a disease that is unfairly treated, because we really felt for, in our skin.” (UK FG)

Participants expressed a desire to support colleagues with long term conditions and reflected on ways in which the workplace might better support people with health conditions, whilst also recognizing the complexities surrounding practices, such as home-working, which have the potential to further isolate and differentiate employees. Participants drew on their own experiences of feeling isolated or being able to share their experiences during In Their Shoes to highlight the therapeutic value placed on social support.

*“The big discovery for this experience is the, the key part of the community, because this time I was not alone with my desk but in a group. And to, to talk about my disease with my colleague I …I think it’s very important, and that’s changed my mind about patient associations.” (France FG*)

#### Organizational Connectivity

“It increases my motivation for my daily job. I do now feel more involved about our mission” (Switzerland).

Although a less dominant theme, a substantive number of participants reported feeling greater pride in working for their company and increased emotional connection to the organization and its mission to serve patients (“the emotional grab wasn’t always there,” United Kingdom). This was particularly evident in those that had more distal, non-patient facing roles.

“*I don’t really have an insight on what’s really going on outside of my department… And I actually felt blessed to be able to work for a pharmaceutical company. I know I’m not a huge part of it, but I’m still part of the people who makes it happen. And I, going home on, on the Wednesday evening, I nearly kind of, nearly cried, because thinking oh God, I’ve got the best job in the world” (UK FG).*

“I am proud that Takeda develops and market IBD medicine for IBD patients” (Japan).

Participants described several mechanisms, firstly, the recognition that their job could make a difference to patients’ lives, irrespective of their role. Secondly, greater connection to patients and confidence in integrating their experience into job roles, such as speaking to others about IBD and considering patient-centered outcomes in research and development. Thirdly, through favorable organizational comparisons and respect for the implementation of novel initiatives (“there’s not many companies out there that would do this sort of thing,” UK FG). Finally, participants valued being able to share their experiences with colleagues, thereby increasing collegiality and providing the opportunity for open discussion and reflection on broader issues around patient experience and care:

“The program allowed people to talk more openly about the impact of IBD and even allowed me to have a discussion with a member of staff who suffers from IBD.” (United Kingdom)

## Discussion

This study found that an immersive training program, conducted amongst pharmaceutical company employees, led to increased understanding of and empathy for the lived experience of patients with IBD. We found statistically significant increases pre/post participation on self-rated disease understanding, self-rated connection with and advocacy for people living with IBD, and belief that job role can positively impact others (prosocial job perceptions). Central themes of how it felt to take part demonstrate significant emotional engagement, with activation of both cognitive and affective empathy, and evidence of a progression from lower order to higher order cognitive skills, moving beyond knowledge (taking on facts) to understanding (drawing conclusions). These findings align with other literature evaluating immersive learning and the potential for increasing empathy through simulation experiences. The present study offers opportunities to extend this outside of the body of work focussing on healthcare practitioners and considers the benefits of using this type of learning experience within a workplace setting. The study’s mixed methods approach offers an in-depth perspective on how empathy can propel learners through the experiential learning cycle and how this may activate behavioral change in the workplace.

### Experiential Learning

Participants’ progression through the experiential learning cycle (Kolb, 1984) is evidenced in both the quantitative and qualitative data. The first phase ‘concrete experience’ is characterized by taking on facts. Improvements in self-rated understanding of the different aspects of IBD demonstrates this knowledge acquisition and a more nuanced picture is offered in the qualitative data with participants highlighting the stress of managing the physical aspects of IBD, particularly the frequent toilet visits. In common with IBD patients, food was also a prevalent topic. One participant venting frustration at the dietary limitations imposed by the condition (“I want a doughnut”) echoed findings by Palant et al. (2015) “And then you start to loose [sic] it because you think about Nutella.” The second phase ‘reflective observation’ is richly described in the qualitative data with participants reporting the intensive, exhausting and emotional burden of IBD in detail. This heightened sense of the psychological impact may be a consequence of the program design and the difficulty of simulating the physical symptoms of IBD. It could also be supported by Kolb’s assertion that the second phase is particularly characterized by the need to address any inconsistencies between past experience and new understanding. So it could be that participants, as employees of a pharmaceutical company, had a working knowledge of disease symptoms, but had not previously considered their psychological and social impact. Illustrations of the third ‘abstract conceptualization’ and fourth ‘active experimentation’ phases can be found in qualitative data as participants explore their insights and reflect on how it would feel if they were unable to ‘switch off’ the experience. Participants interpreted their experience readily and hypothesized how they might incorporate learnings into their role. This finding is supported in the quantitative questions reporting greater connection to people living with IBD and the desire to be personal advocates and enhance representation for those living with the condition, and the increase in prosocial job beliefs.

### Participant Engagement

Findings demonstrate a high level of emotional engagement amongst participants. The central narrative was described as challenging and unpredictable. The frequent text messages distracted participants from their work and removed a level of agency, with qualitative responses including frequent mention of a sense of loss of control. The intensity of the experience was widely reported across program elements, provoking strong emotions (shock, horror, and upset). Participants viewed the authenticity of the simulation both positively and negatively: a tension between knowing that the simulation was robust and accurate set against the reality of the emotional and physical burden. This finding is echoed in other simulation evaluations. Some of the text messages within the central narrative contained patient quotes validating a specific scenario. Participants commented positively on this device. The approach of validating a seemingly fictional scenario as the personal experience of a genuine patient is reported elsewhere to be impactful (Corr et al., 2017; Tong et al., 2017; Ter Beest et al., 2018). The durational nature of the experience, and that participants ‘lived with IBD’ and continued with their work and personal lives deepened the understanding of the impact of illness, yet also helped participants look beyond the disease (“we’re always talking about the patient, the patient, the patient … but it’s not, this is someone leading their everyday life,” UK FG). The value of this intersection of ‘patienthood’ (what it means to be a patient) and real life within a durational experience is supported by Corr et al. (2017) who describe the persistency of the simulation as crucial to its success. In common with other empathy-enhancing interventions, (Batt-Rawden et al., 2013) the program was well received; participants reporting the paradox of enjoying something that was not pleasurable.

Evidence in both the quantitative and qualitative data suggest that the interlinking program elements amplified the central narrative and helped to immerse participants, delivering an enriched and heightened sense of disease impact. Multiple delivery mechanisms may open up a single training intervention to different personal learning styles, although this was not evaluated in the present study. Although this training program cannot be characterized as a serious game, qualitative responses indicated that the gamification devices (for example timed challenges) supported engagement. Participant feedback on the individual intervention characteristics showed that the text messages and role plays were most highly valued. Although the evaluation did not directly ask for feedback on a smart phone mobile application as a training delivery device, it is clear from the qualitative data that this facilitated the intervention’s intensity and intrusiveness as participants’ phones became a constant reminder of their disease state. This may point to mobile applications being a useful delivery mechanism for simulation training. Interactions with others, both those within the simulation (role-play actors) and outside (colleagues, family, and friends) were reported as important for reflections on the psychological and social impact. This is supported in both digital and real-life learning scenarios literature. Bearman et al. (2015) found evidence for role-play as a design feature of successful empathy interventions. The kit items overall were ranked third although those relating to the physical symptoms of IBD were described as particularly impactful. Physical items are reported elsewhere to be confronting for simulation participants, for example nursing students required to wear an incontinence pad for 6 h (Karlowicz and Palmer, 2006). The avatar was less consistently valued, although forms of empathetic mirroring are evidenced in other literature (Norris et al., 2014). Whilst the personalized avatars did show signs of stress and illness during intense narrative sections it may be that the customization features were too basic in this application to influence users’ behavior and motivation, as has been demonstrated elsewhere (Yee and Bailenson, 2007).

### Activating Empathy

The use of mixed methods in this study enabled evaluation and exploration of the activation of empathy amongst participants. Results support both cognitive and affective empathy impacts: an intellectual or imagined understanding (cognitive) and emotional reaction (affective). Spreng et al. (2009) describe an overlap and likely shared processes of these two components hence the Toronto Empathy Questionnaire reports a single score representing both components. Empathy scores significantly increased following participation in the training program. The medium effect size seen in this study is larger than comparable studies reported in Batt-Rawden et al. (2013) review, where only small effect size is reported for seven of the 11 studies where data was sufficient to calculate. Cognitive empathy indicators included conceptualizing the biopsychosocial impact of the illness and being able to explore previously unconsidered components of the disease (for example invasive or embarrassing procedures). Affective empathy was particularly evidenced in the qualitative data, with participants using strong and emotive language to describe their connection to the experience of the IBD patient. This was an interesting finding given the discussion in the literature around whether or not affective empathy can be ‘taught’ (Cuff et al., 2016). The qualitative findings highlight the possibility for empathy to act as a catalyst for behavior change, or as an antecedent to innovative problem solving. Participants discussed their motivation to advocate for patients and their desire to challenge everyday prejudices encountered by people living with IBD – and other chronic conditions. Whilst the current study did not assess behavior change, using an experimental paradigm Batson et al. (2002) were able to evidence an empathy-attitude-action model where participants induced to feel empathy for a stigmatized individual allocated increased funds to an agency providing support.

### Workplace Setting

Disease specific simulation is well documented in HCP training where there is a strong evidence base for enhanced connection between patient and practitioner, supporting improved communication, shared decision making and adherence (Kim et al., 2004; Hojat et al., 2011). However, this has not been studied within the pharmaceutical industry workplace, where a focus on patients is a well-recognized organizational goal. The study participants were from different business roles, both those who had more regular contact with patients (e.g., Marketing and Medical), and those in more administrative settings (e.g., IT, Finance). The study results showed no significant differences based on gender or HCP training. This contrasts with some previous studies which have identified gender (Toussaint and Webb, 2005) and HCP status (Charles et al., 2018) as mediators of empathy. The study also explored in more detail the training’s impact on individual’s perception of role and engagement. This was characterized as the opportunity to have a positive impact on others: how often you could do good (frequency), how much good you could do (magnitude) and the range of positive impacts (scope). Participants perceived the positive impact of their role was greater after they had taken part, recording increases in amount, frequency and opportunity. This connection to role beneficiaries has not been previously studied in a simulation context; only actual exposure to genuine recipients has been shown to improve engagement and performance using this measure (Grant, 2008). Additionally, qualitative findings reflected a more emotional connection to corporate culture (‘pride,’ ‘admiration’) and a shift from a biomedical to patient-centered approach.

### Strengths, Limitations and Future Directions

This was a multicentre international evaluation study, although conducted in a single pharmaceutical company. It provides a novel contribution to the immersive learning literature in its focus on a non-medical audience and workplace context. Moreover, it is the first to measure the impact of immersive learning on job role focus and connectivity. The triangulation of quantitative and qualitative approaches enables an in-depth exploration of the intervention process and mechanisms of change and also tentatively suggests that the observed changes in disease understanding and empathy may have longer-term impact on behavior. Results also demonstrate the success of digital immersive learning to enable perspective-taking inside and outside the workplace – enhancing understanding of the biopsychosocial impact of chronic disease. This points to the potential for mobile applications to deliver immersive learning, offering opportunities to extend training to larger audiences in different disciplines.

However, like the majority of studies in this area, the pre–post evaluation design was a limitation. The lack of a control group or comparator intervention limits our ability to draw definitive conclusions about the causal mechanisms of the intervention on outcome variables. Given that the intervention was delivered and evaluated in the workplace, the observed changes may be due to demand characteristics rather than the intervention itself. The depth of engagement and rich examples of insight and empathy evidenced in the qualitative data suggests, however, that this is not the case. The current study focused only on immediate impact of the training program, so was unable to determine actual behavior change or longer term impact of the intervention. Notwithstanding these limitations, the findings suggest valuable potential directions for future research, to investigate whether changes in empathy and understanding of the lived experience of illness are maintained over time and perhaps more importantly, whether they lead to behavioral change, either on an individual or organizational level.

## Conclusion

The study demonstrated that an immersive training program, focussing on the lived experience of illness, led to significant increases in disease understanding, connectivity and empathy toward patients amongst employees of a pharmaceutical company. In addition to increases in patient connection, this study was the first to demonstrate increases in prosocial job perceptions and organizational engagement in a simulation context. These findings align with other literature evaluating immersive learning and the potential for increasing empathy, motivation and connectivity through simulation experiences in both organizational and medical settings.

## Data Availability

Datasets are available on request: The raw data supporting the conclusions of this manuscript will be made available by the authors, without undue reservation, to any qualified researcher.

## Ethics Statement

This study was carried out in accordance with the recommendations of the ethics committee of University of Westminster. All subjects gave written informed consent in accordance with the Declaration of Helsinki. The protocol was approved by the University of Westminster Psychology Ethics Committee (ETH1617-1855).

## Author Contributions

CH and TC designed the evaluation and wrote the manuscript. CH managed participant enrolment and data collection. TC conducted the quantitative and qualitative analysis with input from CH. CH and TC read and approved the final manuscript.

## Conflict of Interest Statement

CH has previously been employed as a consultant during the development of In Their Shoes^®^training intervention. The remaining author declares that the research was conducted in the absence of any commercial or financial relationships that could be construed as a potential conflict of interest.
